# The relationship between the Hippo signaling pathway and bone metastasis of breast cancer

**DOI:** 10.3389/fonc.2023.1188310

**Published:** 2023-05-15

**Authors:** Qinyu Han, Shi Qiu, Huiwen Hu, Wenjing Li, Xiangguo Dang, Xiangqi Li

**Affiliations:** ^1^ Department of Breast Center, The Second Affiliated Hospital of Shandong First Medical University, Tai’an, Shandong, China; ^2^ Department of The First Clinical Medical School, Shandong University of Traditional Chinese Medicine, Jinan, Shandong, China

**Keywords:** breast cancer, Hippo signaling pathway, metastasis, bone metastasis, targeted therapy

## Abstract

Bone is the most common site of metastasis from breast cancer, which is the most prevalent cancer affecting women globally. Bone metastasis from breast cancer severely affects the quality of life of patients and increases mortality. The molecular mechanisms of metastasis, colonization, and proliferation of breast cancer cells in bone are complex and involve the interaction between breast cancer cells and the bone microenvironment. However, the precise mechanism is not clear at present. In recent years, the Hippo signaling pathway has attracted much attention due to its important role in regulating the expression of major effector molecules during tumor development. In particular, studies have found that the mutation and aberrant expression of the core components of the Hippo signaling pathway affect breast cancer cell migration and invasion, indicating that this pathway plays a role in bone metastasis, although the molecular mechanism of this pathway in breast cancer metastasis has not been fully elucidated. In this review, we discuss the function of the Hippo signaling pathway, introducing its role in breast cancer metastasis, especially bone metastasis of breast cancer, so as to lay a solid theoretical foundation for further research and for the development of effective targeted therapeutic agents.

## 1 Introduction

Breast cancer is the most common malignant disease among women worldwide, and the leading cause of cancer-related deaths in patients (1). The detection and treatment of breast cancer have improved due to advancements in imaging technology, surgery, and medical, biological, and pharmaceutical technology. However, despite these improvements, the global incidence of breast cancer continues to rise, affecting younger individuals, and the mortality rate remains high. Importantly, many cancer patients die not because of tumor growth at the primary site but because of tumor invasion or metastasis to other sites. Bone metastasis is the most frequent site of metastases for breast cancer (2, 3). Clinically, bone metastasis often brings great pain to patients and seriously affects their quality of life. Furthermore, upon metastasis of the tumor to the bone, the condition is typically deemed incurable and the overall prognosis is unfavorable (4). At present, various treatments for breast cancer bone metastases do not significantly prolong the median survival of patients. Although great progress has been made in the study of breast cancer metastasis, the specific mechanism of bone metastasis is still unclear. Therefore, the elucidation of the mechanisms underlying breast cancer bone metastasis remains a daunting task.

The Hippo signaling pathway is a highly conserved inhibitory signaling pathway first discovered in *Drosophila melanogaster* that regulates organ development by inhibiting cell proliferation and promoting apoptosis (5). In recent years, the Hippo signaling pathway has been found to be closely related to tumorigenesis and has thus become a new research hotspot (6–8). However, at present, there are few studies on the role of the Hippo signaling pathway in breast cancer metastasis, especially bone metastasis, and the molecular regulatory mechanisms are still unclear. By reviewing the connection between the Hippo pathway and breast cancer bone metastasis, we hope to shed light on the pathway’s crucial roles and provide the groundwork for using it as a target in tumor treatment.

## 2 Overview of the Hippo signaling pathway

The Hippo signaling pathway consists of three parts: upstream active components, core molecules, and downstream effector molecules. The upstream active components include FAT Atypical Cadherin 4 (FAT4, Fat homology), FEMD6 (Ex homology), neurofibroma protein 2 (NF2, Mer homology), and Dachsous1/2 (DCHS1/2). The core molecules include mammalian Sterile 20-like kinase 1/2 (MST1/2, Hippo homolog), salvador family WW domain-containing protein 1 (SAV1), large tumor suppressor kinase 1/2 (LATS1/2, Warts homolog), and MOB kinase activator 1 (MOB1). The downstream effector molecules include yes-associated protein (YAP) and transcriptional co-activator with PDZ-binding motif (TAZ). The transcription-related parts include TEA domain family members (TEADs) (9).

**Figure 1:**
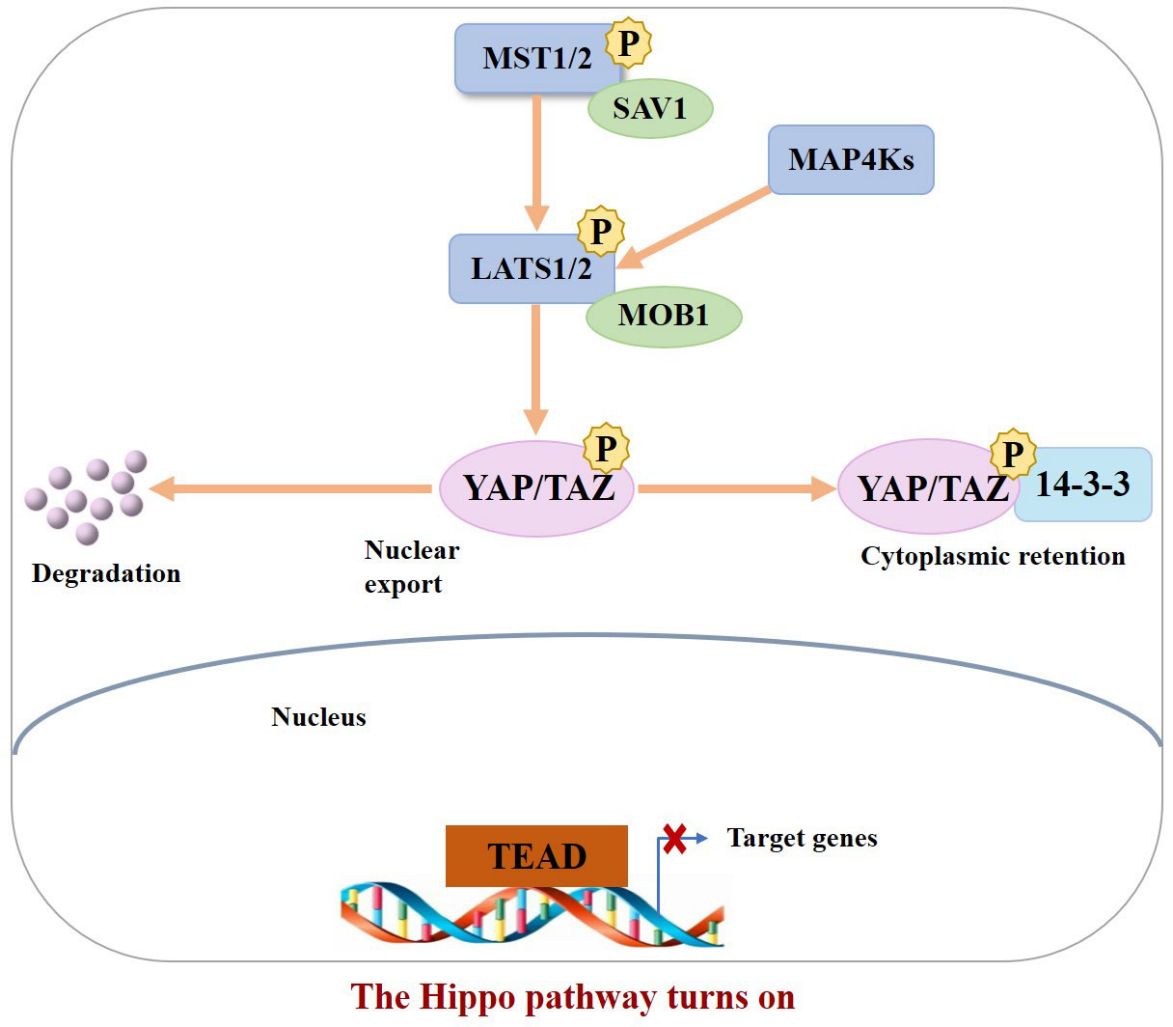
When the Hippo pathway is ON (meaning the kinases are phosphorylated and active), YAP/TAZ are phosphorylated, resulting in their binding to 14–3–3 and cytoplasmic retention as well as degradation.

**Figure 2:**
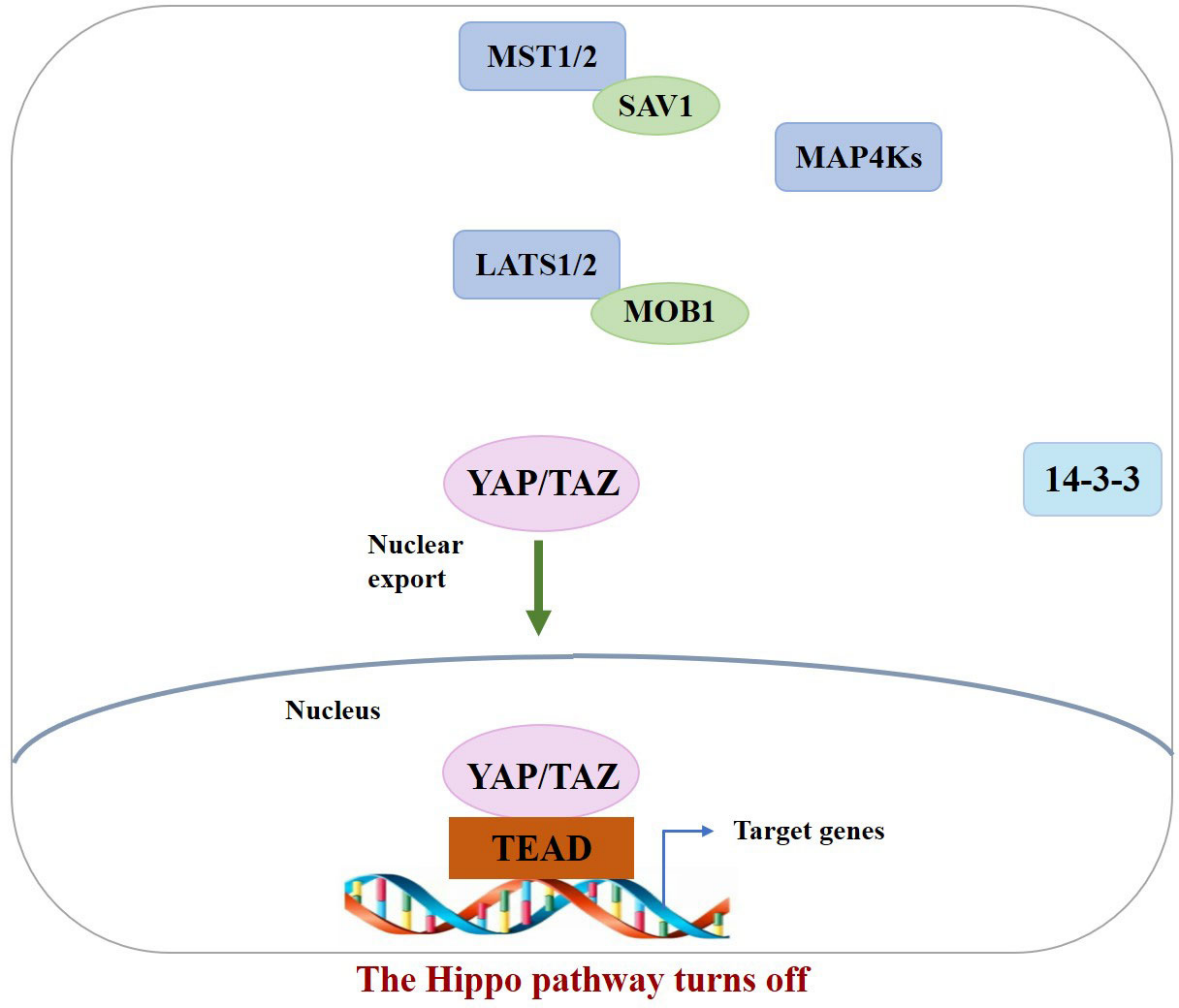
When the Hippo pathway is OFF (meaning the kinases are unphosphorylated and inactive), YAP/TAZ are dephosphorylated and accumulate in the nucleus, where they bind with TEADs.

In the normal physiological state, Hippo signaling pathway activity is strictly regulated. When the Hippo pathway is “turned on,” MST1/2 or MAP4Ks are activated, which then phosphorylate and activate Lats1/2 kinase, forming a complex with the scaffold protein SAV1. It then phosphorylates YAP/TAZ in complex with another scaffold protein, MOB1, which further binds cytoplasmic 14-3-3 proteins and stays in the cytoplasm, resulting in cytoplasmic segregation of YAP/TAZ. It is then degraded by the corresponding protease, ultimately inhibiting cell proliferation (10). In contrast, when the Hippo pathway is “turned off,” YAP/TAZ are translocated to the nucleus and interact with TEAD1–4 to regulate gene expression. This allows the separation of vestigial-like family member 4 (VGLL4) from TEAD1–4, thereby activating gene transcription, ultimately promoting tissue growth and inhibiting apoptosis (11) (**Figures 1**, **2**). If any molecule in this pathway is mutated or abnormally expressed, the balance between cell proliferation and apoptosis will be disrupted, leading to aberrant tissue proliferation or tumorigenesis.

## 3 Roles of the Hippo signaling pathway core molecules YAP/TAZ in breast cancer metastasis

A significant contributor to tumor lethality and breast cancer metastasis is a complex pathological process involving a number of phases that is regulated by numerous genes and signaling pathways (12). YAP and TAZ are the ultimate nuclear effectors of the Hippo signaling pathway and play a role in the metastatic spread of tumor cells and the homing of tumor cells to distant sites (13). Overholtzer et al. (14) were the first to link YAP/TAZ with breast cancer. An increasing number of studies have shown that YAP and TAZ play a very important role in breast cancer metastasis (15, 16).

TAZ levels are elevated in approximately 20% of cancers and drive tumor invasion and metastasis (17, 18). It has been demonstrated that TAZ protein expression levels and activity are upregulated in highly metastatic breast cancers (19). YAP, another core effector molecule in the Hippo signaling pathway, was first found to be a driver of metastasis in breast cancer (20). Studies have shown that YAP expression and activation are positively correlated with lymph node metastasis in breast cancer (21).

### 3.1 Effects of YAP/TAZ on EMT

Metastatic tumor cells exhibit increased motility and invasive capabilities because of epithelial–mesenchymal transition (EMT), a phenotypic conversion that reduces apico-basal pressure and results in the acquisition of mesenchymal properties such as high motility (22). Once YAP/TAZ enters the nucleus, it exerts its oncogenic function in conjunction with bound TEAD, promoting cell proliferation and the expression of EMT-related genes (23). Lei et al. (24) found that TAZ activation induces EMT in normal mammary MCF10A cells. Protease-activated receptor 1 (PAR1) is a G Protein-Coupled Receptors (GPCR) family member involved in cancer cell invasion and metastatic processes. Recent studies have shown that PAR1 acts as a direct transcriptional target of Twist and that it can promote EMT in breast cancer cells by inhibiting Hippo pathway activation by YAP/TAZ (25). In addition, through EMT, tumor cells acquire cancer stem cell features, promoting tumor progression and metastasis. There are scholars who argue that TAZ is essential for maintaining the self-renewal and tumorigenesis of breast cancer stem cells (BCSCs) (15, 26). Bartucci et al. (27) found that the expression level of TAZ in BCSCs was higher than that in differentiated breast cancer cells, and knockout of TAZ in BCSCs inhibited migration.

### 3.2 Effects of YAP/TAZ on invasion and migration of breast cancer cells

Researchers have focused on the role of YAP/TAZ in the invasion and migration of breast cancer cells. A large number of studies have found that YAP/TAZ interacts with other proteins to promote invasion and migration of breast cancer cells. Overexpression of TAZ in MCF10A cells (in which native TAZ expression levels are low) causes morphologic changes characteristic of cell transformation and promotes cell migration and invasion (18). In addition, the TAZ/TEAD complex can induce transcription of amphiregulin (*AREG*), which encodes one of the ligands of the epidermal growth factor receptor (EGFR), to activate an EGFR- but not EGF-dependent signaling pathway that drives cell proliferation and migration, while knockdown of AREG partially attenuates TAZ-dependent migration (28). Interestingly, the above phenomenon is non-cell-dependent, and AREG expressed upon TAZ activation can be secreted extracellularly to activate EGFR in adjacent cells and cause their proliferation and migration.

Cao et al. (29) observed that the leukemia inhibitory factor receptor (LIFR), a known suppressor of breast cancer metastasis, is situated downstream of miR-9 and upstream of Hippo signaling. LIFR was found to be downregulated in breast cancer cells, and LIFR expression exhibited a negative correlation with the likelihood of breast cancer metastasis. Further studies revealed that restoration of LIFR expression in highly malignant tumor cells inhibits metastasis by triggering the Hippo signaling pathway kinase cascade, which leads to phosphorylation, cytoplasmic retention, and functional inactivation of the transcriptional co-activator YAP; in contrast, loss of LIFR in non-metastatic breast cancer cells induces migration, invasion, and metastatic colonization of breast cancer cells through activation of YAP (30). Similarly, deletion of discs large homolog 5 (*DLG5*) in breast cancer cell lines inhibits the Hippo signaling pathway and increases YAP expression in the nucleus, thus promoting breast cancer cell proliferation, migration, and invasion (31). Histone deacetylases (HDACs) have been identified as key regulators of the progression of multiple types of cancer. An et al. (32) demonstrated that HDAC8 promotes the migration of breast cancer cells *in vitro*. Specifically, HDAC8 was found to inhibit the phosphorylation of YAP, which in turn enhanced the migration of triple-negative breast cancer (TNBC) cells. Conversely, silencing YAP was shown to attenuate the HDAC8-triggered migration of TNBC cells.

In addition, it has been found that YAP promotes breast cancer metastasis mainly through interaction with transcription factors such as TEAD (20). It has been shown that DNA damage-activated long non-coding RNA (lncRNA-NORAD) expression is downregulated in breast cancer and its low expression is associated with lymph node metastasis and poor prognosis (33). lncRNA-NORAD expression is significantly inhibited by NORAD upon migration and invasion of breast cancer cell lines. In breast cancer, lncRNA-NORAD expression is downregulated, and the Hippo signaling pathway and the YAP/TAZ–TEAD complexes result in the suppression of NORAD transcription, which in turn promotes tumor metastasis and invasion. Shen et al. (21) showed that focal adhesion (FA) plays a key role in regulating tumor cell motility and invasiveness and that adherent spot kinase (FAK) is a key regulator that promotes FA formation whose upregulation and activation are often associated with breast cancer metastasis and poor prognosis. Interestingly, thrombospondin 1 (THBS1), a stimulator of FAK and a direct transcriptional target of the Hippo signaling pathway, has been observed to increase FAK phosphorylation, thereby enhancing FA kinetics (34). Further studies have shown that YAP activates *THBS1* transcription in a TEAD-dependent manner to induce FAK phosphorylation and promote FA formation, thereby activating tumor cell migration and invasiveness. Recently, it was found that transforming growth factor beta (TGF-β) signaling can synergize with YAP/TAZ–TEAD to regulate breast cancer cell metastasis. Specifically, the TAZ/YAP–TEAD complex binds to pSMAD2/3 to activate a specific pro-oncogenic transcriptional program that induces the expression of the target genes neuronal growth regulator 1 (*NEGR1*) and urothelial cancer-associated 1 (*UCA1*). This consequently promotes the non-anchorage-dependent growth, migration, and tumorigenesis of breast cancer cells (35). The hyaluronan-mediated motility receptor (RHAMM) has been reported to be a breast cancer susceptibility gene with tightly controlled expression in normal tissues but elevated expression in many tumors, contributing to tumorigenesis and metastasis (36). Further studies revealed that YAP/TEAD can bind to the *RHAMM* promoter and control its transcription, which in turn controls the migration and invasion of breast cancer cells (37). Chen et al. (38) found that when the autophagic response was triggered in TNBC cells, YAP was translocated to the nucleus and the expression of the YAP target gene anchor protein repeat domain 1 (*ANKRD1*) was significantly increased, thus promoting the migration and invasion of TNBC cells. Conversely, inhibition of YAP translocation to the nucleus was found to impede the migration and invasion of TNBC cells.

**Figure 3:**
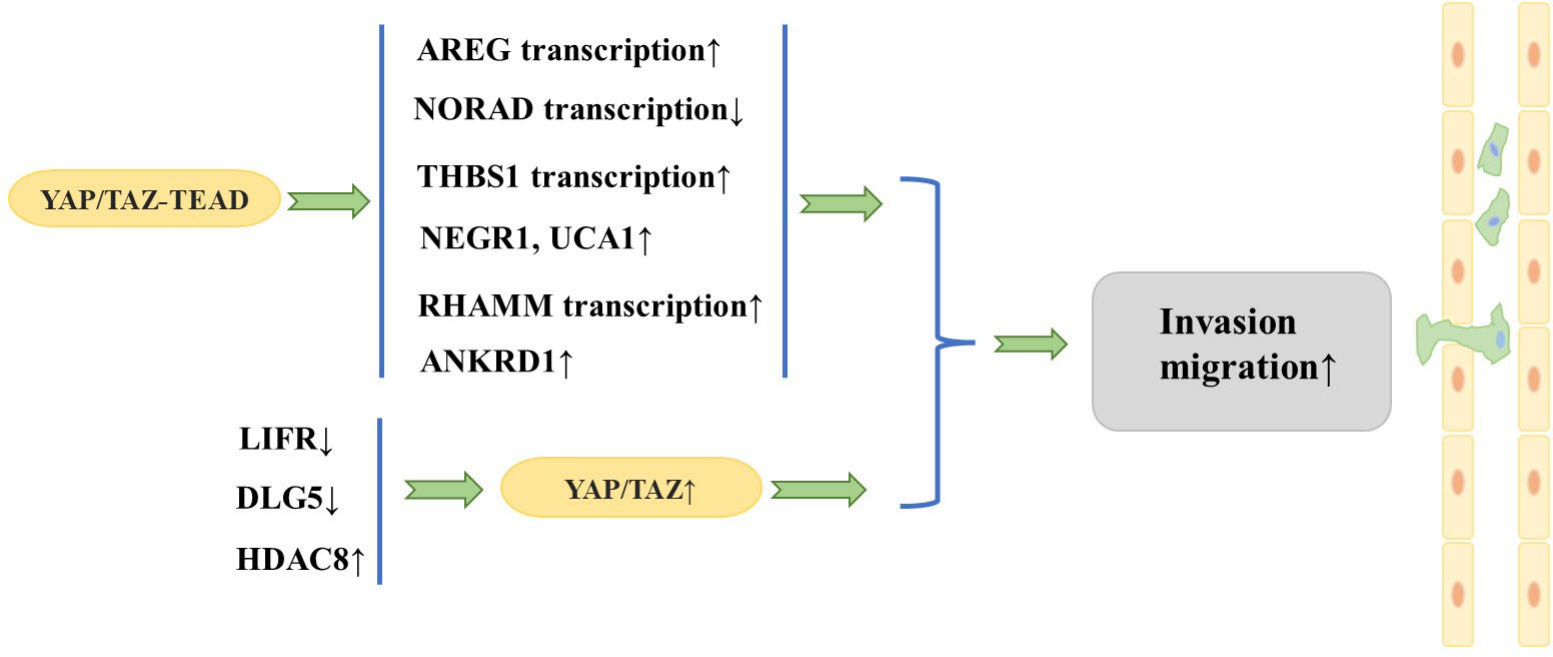
YAP/TAZ act as a promoter in breast cancer cell tumorigenesis.

The aforementioned series of studies together show that YAP/TAZ plays a significant role in fostering metastasis of breast cancer (**Figure 3**). In fact, TAZ has been widely recognized as a cancer-promoting factor, while the function of YAP as an oncoprotein remains a topic of debate. It has been suggested that YAP is able to function as either an oncoprotein or a tumor suppressor, depending on the specific internal environment that is dictated by different subtypes of breast cancer (39). Notably, although YAP and TAZ are highly similar in structure, they are not functionally identical and may play different specific roles mediated by multiple downstream effectors and upstream regulatory molecules (13). Guan et al. (40) showed that LATS1/2 plays a crucial role in sustaining ERα expression through the inhibition of YAP/TAZ, which in turn facilitates the proliferation of ERα^+^ breast cancer cells. In response to this finding, they developed a potent LATS inhibitor, VT02956. By targeting the Hippo pathway, VT02956 represses ESR1 expression and inhibits the growth of ER^+^ breast cancer cells as well as patient-derived tumor organoids (41). To date, it remains to be confirmed by clinical and experimental studies with large samples whether YAP plays the role of oncoprotein or tumor suppressor in the development of breast cancer metastasis.

## 4 Role of the Hippo signaling pathway in breast cancer bone metastasis

Metastasis is defined as the dissemination of neoplastic cells from the primary neoplasm to secondary sites (42). Breast cancer cells disseminate from the original site, invade and translocate into lymphatic vessels and blood vessels through defective areas in the extracellular matrix, and metastasize to distant sites (43). The microenvironment of bones holds various factors that are essential for the proliferation and metastasis of breast cancer cells, thereby creating a conducive environment for the spread of breast cancer to bones. Therefore, bone is the preferred site of breast cancer metastasis, and it has been reported that 70% of metastatic breast cancer patients have bone metastases (44–46).

### 4.1 The Hippo signaling pathway regulates bone metabolism to promote bone metastasis in breast cancer

The balance of the intraosseous environment is maintained by osteoblast-mediated bone formation and osteoclast-mediated bone resorption (47, 48). If there is an imbalance between these two processes, two types of bone metastatic tumors, namely osteolytic and osteoblast metastatic tumors, develop (49). Breast cancer bone metastases frequently arise from bone destruction caused by excessive osteoclast bone resorption; so, the predominant form of bone metastatic tumor is osteolytic (50, 51).

**Figure 4:**
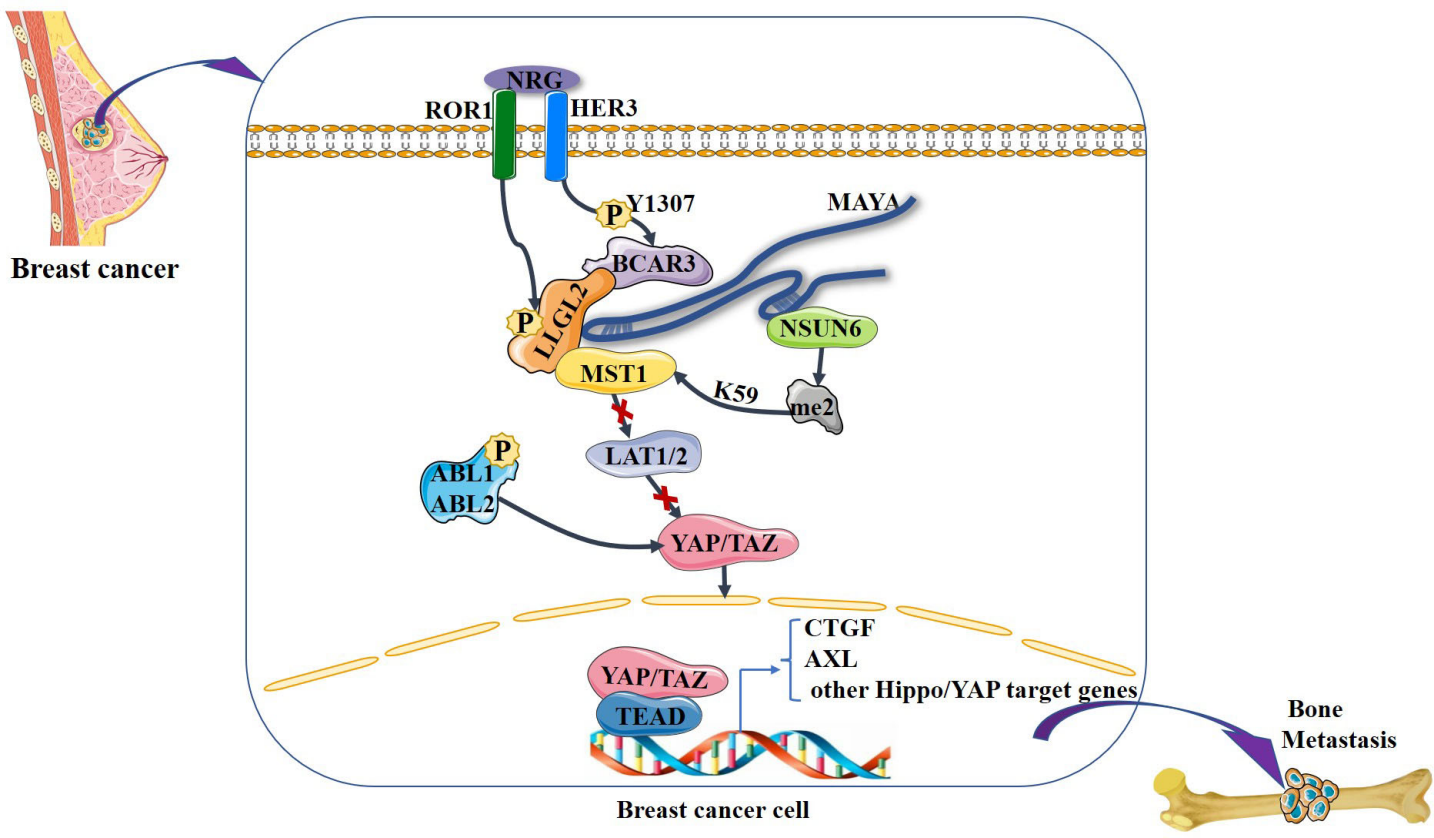
NRG1-induced heterodimerization of ROR1 and HER3 leads to HER3 phosphorylation at Tyr1307, which in turn recruits the LLGL2–MAYA–NSUN6 RNA–protein complex, with lncRNA MAYA binding to both LLGL2 and NSUN6. In this MAYA-mediated mega-RNA–protein complex, ROR1 also phosphorylates LLGL2. Both p-LLGL2 and MAYA are critical for the recognition of Hippo/MST1 by NSUN6. The methyltransferase NSUN6 in the complex methylates Hippo/MST1 at Lys59, leading to a reduction of its kinase activity and hypophosphorylation of LATS1/2 and YAP. Then, YAP is translocated into the nucleus, where it interacts with the transcription factor TEAD to increase the expression of downstream genes such as *CTGF*, thereby promoting bone metastasis. In addition, ABL kinase phosphorylation stabilizes TAZ, which is translocated to the nucleus, where it interacts with the transcription factor TEAD to increase the expression of downstream genes such as *AXL*, thereby promoting bone metastasis.

It is noteworthy that the Hippo signaling pathway has been found to regulate the dynamic balance between osteoclasts and osteoblasts (52, 53). Osteoclasts are the only known cell type capable of resorbing bone matrix, and osteoclast activation is the central cytological mechanism of osteolytic bone metastasis (54). The Hippo signaling pathway is involved in breast cancer bone metastases, primarily by controlling the metabolic homeostasis of bone, as is evidenced by recent findings. Li et al. (55) demonstrated that receptor tyrosine kinase-like orphan receptor 1 (ROR1) promotes invasion, osteoclast differentiation induced by cancer cells *in vitro*, and bone metastasis *in vivo*. ROR1 interacts with human epidermal growth factor receptor 3 (HER3) and can form heterodimers, and further studies have demonstrated that activation of the Hippo–YAP pathway is critical for activating the downstream effects of ROR1–HER3 heterodimers. The specific mechanism involves the recognition of phosphorylated HER3 at Tyr1307 by the SH2 domain-containing protein breast cancer anti-estrogen resistance 3 (BCAR3), which subsequently recruits the adaptor protein lethal giant larvae homolog 2 (LLGL2), allowing the latter to be phosphorylated by ROR1. LLGL2 has previously been reported to play a key role in mediating the cell–cell junction-triggered Hippo signaling pathway (56). Subsequently, the LLGL2–MAYA–NSUN6 RNA–protein complex methylates Hippo/MST1 at the 59th lysine residue (Lys59). This methylation leads to MST1 inactivation, which results in YAP/TAZ activation in tumor cells, inducing osteoclast differentiation and bone resorption, ultimately promoting bone metastasis. In addition, Wang et al. (57) found that high expression of the gene encoding ABL kinase was associated with breast cancer bone metastasis. Subsequent investigations demonstrated that knockdown of ABL kinase resulted in decreased *TAZ* mRNA expression and reduced binding between TAZ and its downstream target AXL, a receptor tyrosine kinase that promotes breast cancer bone metastasis, and breast cancer bone metastasis was inhibited (58). Bartucci et al. (27) also found that nuclear expression of TAZ was significantly higher in bone metastases than in the primary tumor. In these studies, it seems that the Hippo signaling pathway plays a very important role in breast cancer bone metastasis. A variety of factors in breast cancer cells “turn off” the Hippo signaling pathway and YAP/TAZ enters the nucleus to bind to downstream target genes, stimulating the development of breast cancer bone metastasis (**Figure 4**).

In addition, hypoxia-activated HIF-1 in bone marrow may promote the formation of osteolytic bone metastases by inhibiting osteoblast differentiation and promoting osteoclastogenesis (59). This process may be related to the Hippo signaling pathway regulating breast cancer bone metastasis. It has been shown that trans-activation of HIF-1 is regulated by the interaction of E-cadherin and Hippo signaling pathway effectors (60). Research has revealed that certain genes, such as *TFF3*, *EGLN1*, *SNAI1*, *MMP9*, *TGFB3*, *SLC2A3*, and *CTGF*, are subject to direct regulation by hypoxia (61). Under hypoxic conditions, the binding of TAZ to the *CTGF* promoter increases, resulting in a HIF-1α-dependent increase in *CTGF* mRNA levels (62). Hypoxia enhances the co-localization of TAZ and HIF-1α in the nucleus of human 1833 cells while interfering with the DNA-binding activity of the HIF-1 dimer complex (61, 63). In conclusion, HIF-1α interacts with TAZ and stimulates breast cancer bone metastasis in a hypoxic microenvironment. It is worth noting that oxidative stress (OS)/COX-2 may be the molecular link between hypoxic stimulation, the Hippo pathway, and the transcriptional regulator Snail. Blocking COX-2 downregulates the expression of HIF-1a and Snail in the nucleus of hypoxic 1833 cells, and then, TAZ is phosphorylated by interacting with LATS. The nuclear localization of LATS promotes TAZ translocation in the cytoplasm, mediates TAZ phosphorylation and degradation, inhibits TAZ entry into the nucleus, regulates TAZ transcriptional co-activation, and prevents tumorigenesis (63). Therefore, hypoxia and HIF play an important role in breast cancer bone metastasis, and they may be important factors regulating the EMT status of primary and secondary tumors. Based on the aforementioned studies, it appears that YAP/TAZ inhibitors and COX-2 inhibitors hold promise as novel avenues for drug development, providing new solutions to prevent tumor progression, reverse the tumor microenvironment, and break the malignant cycle.

### 4.2 The Hippo signaling pathway mediates other signaling pathways to regulate breast cancer bone metastasis

The Hippo signaling pathway influences the homeostasis of bone-metabolizing cells by regulating a complex network of core components; moreover, it maintains the cellular microenvironment of bone metabolism by interacting with multiple signaling pathways and plays a regulatory role in bone metastasis of tumors (64). β-Catenin is a key transcriptional regulator downstream of the Wnt signaling pathway that regulates the expression of osteogenic proteins such as RUNX2 and Osterix. When the Wnt signaling pathway is inactivated, β-catenin is phosphorylated and degraded in the cytoplasm under the action of a degradation complex. YAP/TAZ is involved in the formation of this degradation complex. Conversely, when the Wnt signaling pathway is activated, its ligands inhibit the function of the degradation complex, and β-catenin enters the nucleus and interacts with the YAP/TAZ–TEAD complex to jointly regulate the expression of downstream target genes (65, 66). Studies on breast cancer with bone metastasis have found that bone metastasis tumor cells can not only enhance the function of osteoclasts and interfere with normal bone remodeling but can also inhibit osteoblasts and prevent new bone formation (67). The above processes regulate the proliferation and differentiation of osteoblasts through interactions between the Wnt signaling pathway and the Hippo signaling pathway, thus affecting bone metastasis. Notably, in recent years, Snail and Slug have been shown to be closely associated with the pluripotency of mammalian cells and the self-renewal of stem cells, making it possible to explore the association of Snail and Slug with the Hippo signaling pathway and their role in the regulation of cellular function and metabolism (68, 69). Tang et al. (70) found that Snail/Slug interacts with YAP/TAZ, controlling the self-renewal and differentiation of bone marrow mesenchymal stem cells, thereby affecting bone development and formation. Both Snail and Slug can form a binary complex with YAP or TAZ, which then regulate the expression of downstream target genes, such as alkaline phosphatase (*ALP*), *RUNX2*, and *Osterix (* 71). So, this process promotes osteogenesis.

**Figure 5:**
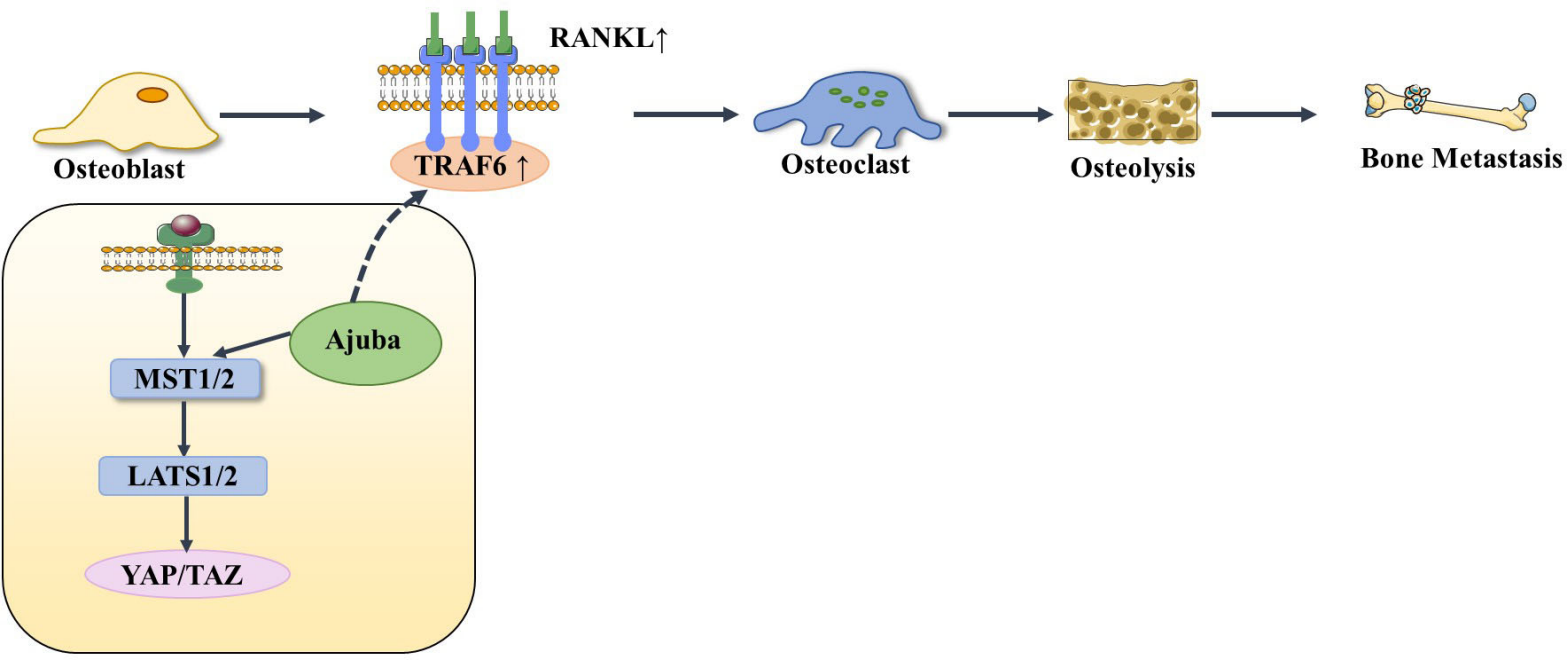
When RANKL is activated, it recruits more TRAF6 under the action of Ajuba, stimulating osteoblasts to differentiate into osteoclasts, thereby causing osteolysis and bone metastasis.

**Figure 6:**
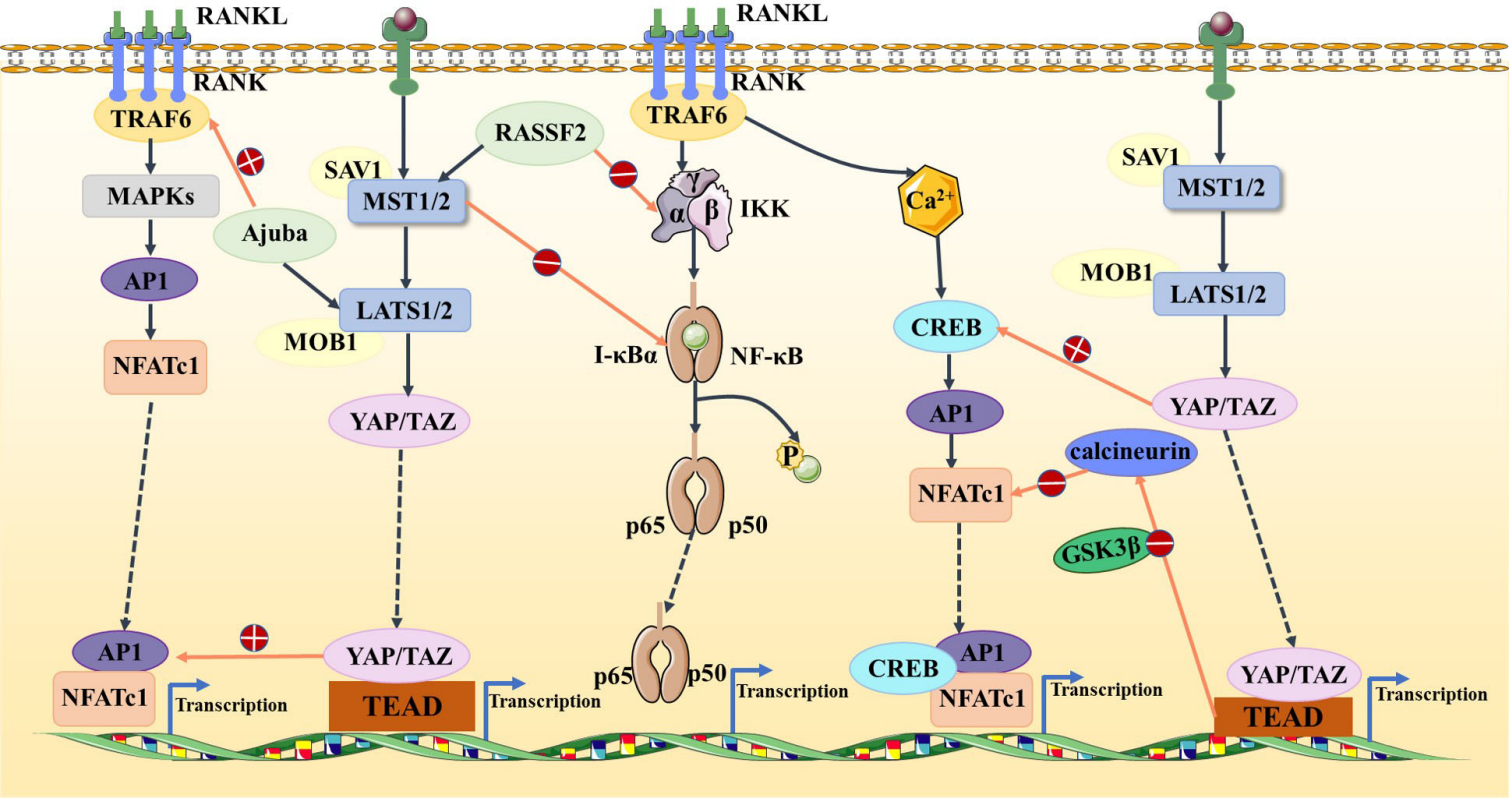
In the Hippo and NF-κB signaling pathways, RASSF2 and MST2 suppress IKK and IκBα activities, respectively, blocking the NF-κB signaling pathway. In the Hippo and MAPK signaling pathways, Ajuba activates TRAF6 and the YAP/TAZ–TEAD complex activates AP1. In the Hippo and calcium signaling pathways, YAP activates CREB and TEAD-dependent downregulation of calcineurin activity, thus inhibiting NFATc1.

The Hippo signaling pathway has been reported to be associated with the RANKL/RANK signaling system. It is well known that the RANKL/RANK signaling system is associated with almost every step of breast cancer development, from primary tumorigenesis to the establishment of secondary bone tumors (55). Osteoclast-mediated bone resorption is a crucial and initial step in the development of osteolytic lesions in breast cancer (67). The interplay between the receptor activator of nuclear factor-kappa B (RANK) and its ligand (RANKL) also plays a significant role in the development of osteolytic lesions of breast cancer (72). At present, research on its signaling pathways regulating osteoblast differentiation is mainly focused on RANKL-related signaling pathways. Specifically, increased RANKL levels lead to hyperactivation of osteoclastogenesis and bone resorption, paving the way for metastatic clones to invade the bone (43, 73). Tumor necrosis factor receptor-associated factor 6 (TRAF6) is an important component of the RANKL/RANK signaling system, which activates downstream signaling cascades and is one of the critical factors for osteoclast activation. Ajuba, a member of the Hippo signaling pathway, interacts with TRAF6 and positively influences TRAF6 activation, thereby regulating downstream factors that trigger osteoclast activation and bone resorption (74). This leads us to hypothesize that when more TRAF6 is recruited in the presence of Ajuba after RANKL is activated, it triggers massive osteoclast activation, which leads to osteolysis and thus promotes bone metastasis (**Figure 5**). Studies have shown that downstream of MST and LAST in the Hippo signaling pathway, the transcriptional co-activators YAP and TAZ bind to members of the TEAD family of transcription factors to regulate the expression of downstream target genes, such as connective tissue growth factor (*CTGF*/*CCN2*) and cysteine-rich protein 61 (*CYR61*/*CCN1*), and the junctional CTGF/CCN2 complex plays an important role in promoting osteoclast formation and osteolytic metastasis in breast cancer (75). In research on the effect of ectodermal-neural cortex 1 (ENC1) on radioresistance in breast cancer cells and showed that overexpression of ENC1 promoted intranuclear translocation of YAP/TAZ, enhanced the expression of GLI1, CTGF, and FGF1, and promoted the progression of breast cancer cells to bone and brain metastasis (76). Interestingly, CTGF also binds to osteoprotegerin (OPG) and RANK to activate the NF-κB signaling pathway and promote osteoclastogenesis, suggesting that the binding of YAP to TEAD family transcription factors mediates the execution of the osteoclastic program (77, 78). In addition, OPG acts as a pseudoligand for RANKL and competitively binds RANKL to avoid osteoclastogenesis and thus protects bones. Binding of TRAF6 to the cytoplasmic region of RANK leads to the activation of NF-κB, which is then translocated to the nucleus. The translocated NF-κB, in combination with P65, results in the interaction of the nuclear factor of activated T cell 1 (NFATc-1) and c-Fos. This interaction contributes to the transcription and expression of osteoclast genes, ultimately inducing the formation of mature osteoclasts. Notably, in MST2-deficient osteoclast precursor cells, the NF-κB signaling pathway is in an activated state, and the RANKL receptor increases NFATc1, Acp5, and OSCAR expression, ultimately promoting osteoclastogenesis; in contrast, when osteoclast precursor cells overexpress MST2, the NF-κB signaling pathway is inhibited and osteoclastogenesis is also inhibited (79). In addition, the Ras-association domain family (RASSF), an upstream regulatory protein of MST, possesses a SARAH domain that can bind to it. RASSF1A, RASSF2, and RASSF8 can inhibit the transcriptional activity of the NF-κB signaling pathway (80, 81). Song et al. (82) established an RASSF2^-/-^ mouse model and found that RASSF2 defects caused developmental delay in mice and observed a severe osteoporosis phenotype. Moreover, RASSF2 deficiency leads to the overactivation of NF-κB during osteoclast differentiation. The observed negative correlation between MST2 expression and osteoclastogenesis implies that RASSF2 may play an essential role in osteoclast formation by binding to the Hippo signaling pathway protein MST2 and interacting with the RANKL-mediated NF-κB signaling pathway. It has been reported that activation of the NF-κB signaling pathway, as well as Jun N-terminal kinase (JNK), the calcium signaling pathway, MAPK, and other signaling pathways, can be mediated by the binding of RANKL to RANK (64, 83). These pathways are known to play a key role in the development and activation of osteoclasts. Thus, it seems that the interactions between the abovementioned signaling pathways and the Hippo signaling pathway affect the activation of osteoclasts (**Figure 6**). However, the extent to which this interplay influences bone metastasis in breast cancer remains unclear and warrants further investigation.

## 5 Discussion and conclusion

Bone metastases from breast cancer are associated with a mean survival period of 2–3 years after diagnosis, and are responsible for bone pain and skeletal-related events that can significantly impact the quality of life of the affected individuals (84, 85). Breast cancer bone metastasis is a complex process involving interdependent stages that cannot be attributed to a single mechanism, and its mechanism of action requires further investigation. The interaction between the bone microenvironment and tumor cells is an important cause of bone metastasis, and osteoclasts in the bone microenvironment play an important role in osteolytic bone metastasis (86). A variety of molecules and signaling pathways are involved in regulating the process of bone metastasis and, ultimately, the formation of osteolytic lesions (87). Considering the limited effectiveness of currently applied therapies, it is crucial to understand the mechanisms of breast cancer bone metastasis, to explore new potential targets, and to develop effective therapeutic regimens. This review details the role of the Hippo signaling pathway in breast cancer metastasis, with a specific emphasis on breast cancer bone metastasis.

The Hippo signaling pathway plays a role in regulating cell growth and suppressing tumorigenesis through a series of enzymatic kinase chain reactions. Blockage of this pathway can lead to tumorigenesis. Studies have found that YAP/TAZ, a downstream effector of the Hippo signaling pathway, plays an important role in breast cancer metastasis. TAZ is widely recognized as an oncoprotein; however, further studies are required to determine whether YAP is an oncoprotein or a tumor suppressor. Most studies have suggested that YAP is an oncoprotein and that overexpression of YAP in the nucleus promotes breast cancer progression and metastasis. The mechanism of action of the Hippo signaling pathway in breast cancer bone metastasis is mostly related to osteoclast activation and osteolysis. The Hippo signaling pathway affects breast cancer bone metastasis by regulating the bone microenvironment. The core components of this pathway bind to downstream factors to activate downstream target genes. Recent studies have established that YAP binds to TEAD to activate CTGF, which promotes osteoclast activation and breast cancer metastasis. Interestingly, the Hippo signaling pathway in breast cancer cells can also affect bone metabolism through the RoR1–Her3–lncRNA signaling axis and ABL kinase, thus regulating breast cancer bone metastasis. In the bone marrow hypoxic microenvironment, HIF-1α interacts with TAZ to promote breast cancer bone metastasis. In addition, the Hippo signaling pathway interacts with the Wnt signaling pathway, the Snail/Slug signaling pathway, and RANKL/RANK-related pathways to regulate the bone microenvironment, which further affects bone metastasis. Many studies have reported that the interaction between the Hippo signaling pathway and RANKL/RANK-related pathways can regulate osteoclast activation and bone resorption, but the precise regulatory mechanism is not fully understood. We can only postulate that the Hippo signaling pathway promotes osteoclast activation through the RANKL/RANK signaling system, which in turn promotes breast cancer bone metastasis. However, the precise role of the Hippo signaling pathway in the promotion of bone metastasis remains to be elucidated, which calls for further investigation. The current treatments available for patients with bone metastases from breast cancer are based on disrupting inappropriate signaling between breast cancer cells and cells in the bone microenvironment by using bisphosphonates and denosumab (43). Unfortunately, these medications have severe adverse effects and also impede normal bone healing by disrupting signaling between cancerous and non-cancerous cells in the bone microenvironment. Therefore, future studies should concentrate on the identification of key upstream regulatory factors of molecular signaling pathways in the bone microenvironment regulated by breast cancer cells. The Hippo signaling pathway may offer new possibilities for the development of effective targeted therapeutic agents.

## Author contributions

QH wrote this manuscript. QH and SQ drew the pictures in this paper. In addition, HH, WL, and XL revised the manuscript. All authors contributed to the article and approved the submitted version.
